# Mechanical Properties Distribution within Polypropylene Injection Molded Samples: Effect of Mold Temperature under Uneven Thermal Conditions

**DOI:** 10.3390/polym9110585

**Published:** 2017-11-07

**Authors:** Sara Liparoti, Vito Speranza, Andrea Sorrentino, Giuseppe Titomanlio

**Affiliations:** 1 Department of Industrial Engineering, University of Salerno, via Giovanni Paolo II, 132, 84084 Fisciano, Italy; sliparoti@unisa.it (S.L.); gtitomanlio@unisa.it (G.T.); 2 Institute for Polymers, Composites and Biomaterials (IPCB-CNR), Via Previati, 1/C, 23900 Lecco, Italy; andrea.sorrentino@cnr.it

**Keywords:** indentation, Harmonix AFM, polymer morphology, mechanical properties

## Abstract

The quality of the polymer parts produced by injection molding is strongly affected by the processing conditions. Uncontrolled deviations from the proper process parameters could significantly affect both internal structure and final material properties. In this work, to mimic an uneven temperature field, a strong asymmetric heating is applied during the production of injection-molded polypropylene samples. The morphology of the samples is characterized by optical and atomic force microscopy (AFM), whereas the distribution of mechanical modulus at different scales is obtained by Indentation and HarmoniX AFM tests. Results clearly show that the temperature differences between the two mold surfaces significantly affect the morphology distributions of the molded parts. This is due to both the uneven temperature field evolutions and to the asymmetric flow field. The final mechanical property distributions are determined by competition between the local molecular stretch and the local structuring achieved during solidification. The cooling rate changes affect internal structures in terms of relaxation/reorganization levels and give rise to an asymmetric distribution of mechanical properties.

## 1. Introduction

Understanding the effect of the process on the internal morphology of polymeric artifacts and its relationship with the final mechanical properties is the key-point for a correct prediction of reliability, performance and durability of a product. The complexity of the internal features, developed in the micro and sub-micro scales during common processing of thermoplastic polymers, requires additional studies on the relationships between morphology and properties. Consistently, characterization tests on micro and sub-micro scales, such as micro and nano indentation, are emerging as new approaches to provide quantitative information about the mechanical properties and morphology of polymeric materials [1,2]. The ability to measure properties on the nanometric length-scale is particularly important for objects such as molded parts in which the localized internal structures can have a significant impact on the bulk properties [3,4,5,6,7,8].

Recently, a multiscale mechanical characterization of injection-molded samples was performed by combining dynamic mechanical analysis, micro Indentation, and HarmoniX Atomic Force Microscopy tests [9,10]. Those results show that the molded samples present a complex multilayer morphology in which, starting from the sample surface, less organized structures (globules), characterized by mechanical properties similar to the quiescent amorphous phase, coexist with well-organized and oriented structures (fibrils), characterized by a high mechanical modulus. Intermediate behavior was found in the central part of the sample, where well developed spherulitic structures are characterized by a lower level of orientation [11,12].

The morphology developed in the molded parts can be highly dependent on imperfect or wrong processing conditions. They can be due to problems related to a combination of poor material characterization, imperfect tooling design, and inadequate control of the processing variables [13,14,15,16]. Knowing how intentionally created molding defects impact on the final product quality can help to recognize and avoid them in real conditions. Probably, among other parameters, the mold temperature is the most critical parameter to be monitored and controlled [17,18]. Despite the extensive studies, a clear correlation between the product properties and the mold temperature is not yet completely established. The situation becomes still more complex when a complete and accurate temperature control is difficult or even impossible. As a matter, non-controlled asymmetrical temperature fields are quite common when geometrical constraints, non-balance (or failure) of the cooling systems, and local overheating are present. In some special cases, high-temperature gradients are imposed by specific production requests such as micro-injection, in-mold labeling and over-molding [19,20]. Regardless of the specific reason for the non-uniform temperature, the results may give rise to severe problems such as inhomogeneous filling, low cycle repeatability, warpage, a lack of part performances and poor surface finishing [21,22]. The uneven temperature field may produce a sample with strong mechanical unbalancing with the possibility of warpage and the formation of cracks.

Liparoti et al. [12,23] reported the effect of induced asymmetrical fast surface temperature evolution on the morphology of injection molded isotactic polypropylene (iPP) samples. They showed a strong effect of two factors on the morphology of the final part: increasing the cavity surface temperature and varying the temperature pulse duration [24]. Despite the continuous technological interest in this subject, only a few papers have tried to correlate the complex internal morphology produced in these conditions with the local mechanical properties within the molded parts.

The objective of this work is to provide an experimental morphological and mechanical characterization of molded samples obtained in highly asymmetrical mold temperature conditions. The mechanical properties along the sample thickness have been correlated to the mold temperature condition adopted. The correlation among processing conditions, mechanical properties and internal morphology of the moldings are also analyzed and discussed.

## 2. Materials and Methods

Thin bars of isotactic polypropylene (iPP) with a rectangular section were obtained by injection molding, using a mold cavity that can impose high temperature gradients in a very short time. The iPP adopted for the experiments was supplied by Montell (Ferrara, Italy), now Lyondell Basell Industries. It is a commercial grade (tradename Moplen T30G). Moplen T30G is a general purpose homopolymer for extrusion/molding applications, with a melt flow index of 3.6 (ASTM D1238/L). The molecular weight distribution was determined by a size exclusion chromatography, with weight-average molar mass *M*_w_ of 376,000 g·mol^−1^, polydispersity index *M*_w_/*M*_n_ of 6.7, and a meso pentads content 87.6%.

**Figure 1:**
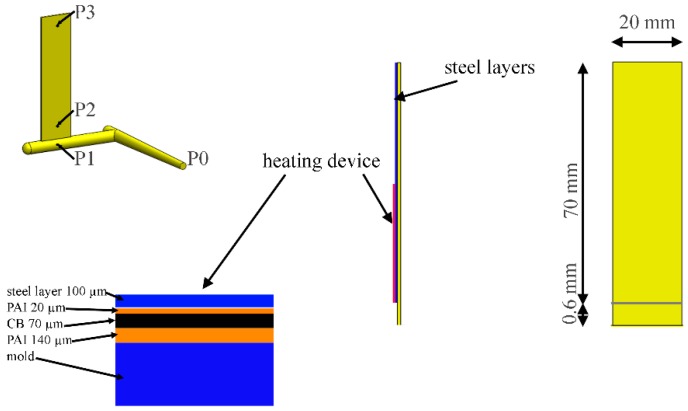
Sketch of the cavity geometry and of the heating device adopted. Positions P0 ÷ P3 where the pressure evolutions were measured during the injection molding tests are also indicated. (PAI = poly(amide-imide) insulating layer; CB = electrical conductive layer, made of carbon black loaded PAI).

Figure 1 shows the geometry of the samples and the sketch of the heating device. In particular, the mold has a gate 1 mm thickness and a rectangular cavity of length *L* = 70 mm, width *W* = 20 mm, and thickness *S* = 1 mm.

The injection molding tests were carried out with a 70-ton Canbio reciprocating screw, injection-molding machine (Negri-Bossi, Cologno Monzese, Milan, Italy). The experiments were performed with an average volumetric flow rate of 3 cm^3^·s^−1^ (the cavity filling time was about 0.5 s), a melt injection temperature of 220 °C and a mold temperature of 20 °C. A holding pressure of 300 bar was adopted with a holding time of 2 s. The pressure was measured in four different positions along the flow path by means of piezoelectric pressure transducers. In particular, one transducer is located in the injection chamber, P0, one just before the gate, P1, and two in the cavity, P2 and P3 (20 and 50 mm downstream from the gate). Their locations are indicated in Figure 1. As also shown in Figure 1, a thin heater is located below the cavity surface and it is protected by a thin steel layer (100 μm). The thin heater was adopted for quickly and accurately controlling the temperature on one side of the cavity (on the moving plate of the mold). A detailed description of the heating device is reported elsewhere [25]. The melt temperature in position P2 was measured with a temperature probe located on the protective steel layer.

The molding conditions adopted in the tests considered in this work are summarized in Table 1. During the injection molding tests, the heating device was supplied by a constant electrical power, *P*_e_. The heating device was activated for two seconds, *t*_a_, before the local contact with the polymer in position P2, where pressure and temperature were measured. After that, the electrical power was supplied for additional heating times, *t*_h_, 0.5 s, 8 s and 18 s. The asymptotic temperature, *T*_level_, reached on the cavity surface when the heater is supplied with 7 W/cm^2^, for longer heating time, is 120 °C. For comparison, a non-conditioned experiment, T_off, was performed without activating the heater (in this case *T*_level_, is the ejection temperature, 28 °C).

### 2.1. Sample Preparation

The specimens were cut in position P2 from the cross section of the sample and carefully polished with a predefined procedure [9]. For the Atomic Force Microscopy (AFM) characterizations, an additional chemical etching procedure was carried out on the specimens in order to eliminate local micro deformation induced by the cutting procedures. In particular, the Basset’s etched procedure [12,23,26] was adopted to prepare selected samples to the AFM analysis. A solution of potassium permanganate, in a mixture of 10:4:1 volumes of concentrated sulphuric acid, orthophosphoric acid and distilled water (1 g of potassium permanganate in 100 mL of mixture), was adopted as etchant. A 2 h period of etching at room temperature was generally sufficient to reveal the surface topography.

### 2.2. Optical Microscopy Tests

In order to gather information on sample morphology, cross section slices with 0.1 mm thickness were cut from injected samples by means of a Leica slit microtome in position P2. These samples were observed in Polarized Optical Microscopy (POM) with an optical microscope (model Olympus BX41) equipped with a digital camera by using a magnification level of 20X. Micrographs of the slices were taken so that flow direction was rotated of 45° with respect the analyzer direction.

### 2.3. Indentation Tests

Indentation tests were performed with a Nano Test™ Platform (Micro Materials Ltd., Wrexham, UK). Tests were carried out with an initial load of 0.02 mN, a load rate of 1 mN/s and a maximum load of 120 mN held for 60 s. A three-sided Berkovich pyramidal diamond tip with a radius of about 100 μm was adopted to indent the surface of the sample. Elastic modulus were evaluated from each imprint, directly by Nano Test™ Platform software 4, according to the Oliver and Pharr method [27].

### 2.4. AFM Analysis

AFM analyses were performed by a NanoScope MultiMode V scanning probe microscope (Veeco, Santa Barbara, CA, USA) equipped with HarmoniX tool. Tests were performed with HMX probe silicon cantilevers (Bruker, Billerica, MA, USA) with nominal radii of c.a. 10 nm. The cantilever oscillation is composed by two different movements, torsional and vertical. These movements have different frequencies, in particular the amplitude frequency of torsional movement is higher than the tapping frequency [28,29]. The reconstruction of sample morphology is due to the vertical movements in standard tapping mode, whereas, the reconstruction of elastic modulus maps is due to the tip sample force interactions during the torsional movement [28]. Lastly, the AFM elastic modulus distribution is obtained averaging the elastic moduli on windows of 20 × 20 μm by the Nanoscope software version 7.30.

HarmoniX measurements were done in air. Cantilevers were calibrated using a standard polystyrene/low density polyethylene (PS/LDPE) sample. The adopted vertical frequency was 44 kHz and the torsional frequency was 989 kHz. The cantilever vibration free amplitude was of 750 mV in air. The force level was modulated adopting the amplitude set point. The amplitude set point was used for feedback control, as a reference value; in particular ca. 60% of the free amplitude was selected. Imaging was performed with 0.5 Hz scan rates, considering 20 harmonics.

The calibration method reported by Gojzewski et al. [4] allows evaluating the deflection sensitivity and cantilever spring constants that were 360.6 nm/V and 1.58 N/m respectively.

Image processing and data analysis were performed with the NanoScope software version 7.30 and NanoScope Analysis version 1.20. The NanoScope software gives elastic modulus maps by elaborating HarmoniX AFM imaging through Derjaguin-Muller-Toporov (DMT) model [30].

## 3. Results

### 3.1. Molding Experiments

**Figure 2:**
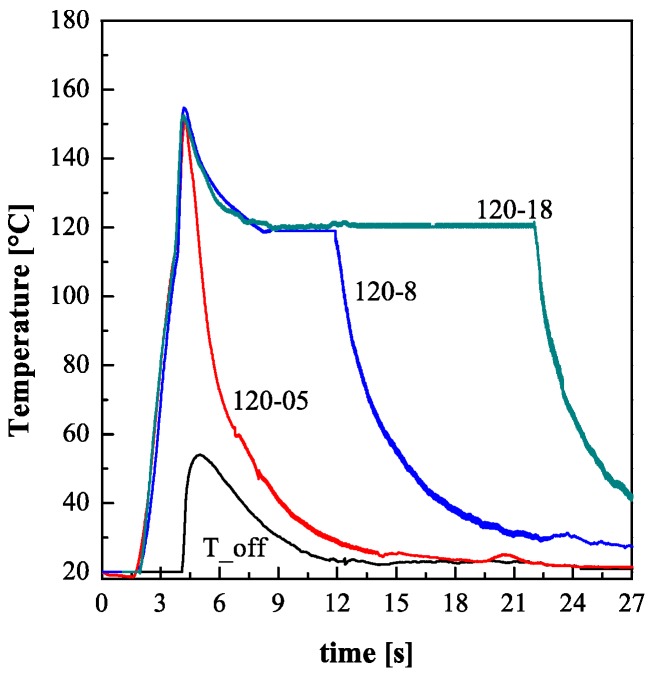
Temperature evolutions measured during tests reported in Table 1.

An example of the temperature evolutions recorded during the molding experiments is reported in Figure 2. In the same figure, the temperature evolution recorded for the experiment T_off is also reported. The activation of the electric heater starts two seconds before the melt reaches position P2 (*t* < 3.7 s), during such a time the temperature rises up to about 120 °C. At this point, the temperature increase up to about 150 °C, is due to the contact of the hot melt with the cavity surface. Obviously, the temperature recorded for the experiment T_off only shows the temperature increase due to the hot melt contact. In all cases, after a peak the temperature starts to decrease down to the selected *T*_level_, i.e. 120 °C for tests performed with the heater activated and 28 °C for the experiment T_off. For the tests performed with the heater activated, soon after the heater deactivation, the temperature decreases quickly to 28 °C. The cooling phase duration depends only slightly on the duration of the heating stage.

**Figure 3:**
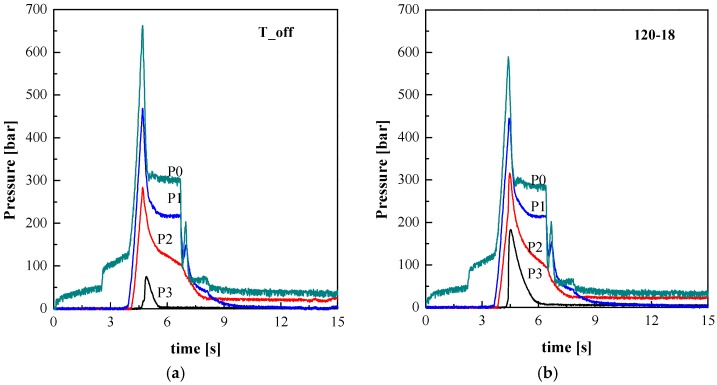
Pressure evolutions measured in different positions along the flow path for the tests (**a**) T_off and (**b**) 120-18.

Figure 3 shows the pressure evolutions recorded in different positions along the flow path of two selected experiments, T_off and 120-18. After the filling stage (that ends after 4.5 s), the pressure undergoes a sudden increase upwards, reaching a sharp peak followed by a quick decrease toward the holding value. The peak in the pressure evolutions is higher in the T_off experiment because the pressure required to fill the cavity is higher at ambient temperature. As soon as the holding pressure in P0 is released (*t* = 6.5 s), the pressure in P1 quickly reduces. Half a second after the holding pressure release, the screw rotates again and moves back to its starting position, applying a back-pressure for about 0.5 s (batching step). As the melt viscosity in the sprue and runners is low enough, the pressure evolution in P1 follows the pressure in P0 showing a similar back-pressure spike. On the opposite side, the viscosity in the cavity is so high to prevent any back-pressure effect on the pressure evolutions in position P2 and P3.

Apart from some minor differences, the high temperature reached on one side of the sample surface seems to have only marginal effect on the pressure evolution recorded on the unheated side. A similar finding was described in previous works [12]. The pressure evolution in a certain position depends on the temperature distribution along the flow path. Comparing the pressure evolution of the T_off test with the 120-18 test it is evident that in the T_off case the pressure drop between position P2 and P3 is significantly higher. This is due to the fact that the melt cools down faster since it is in contact with a colder surface.

### 3.2. Optical Morphology

**Figure 4:**
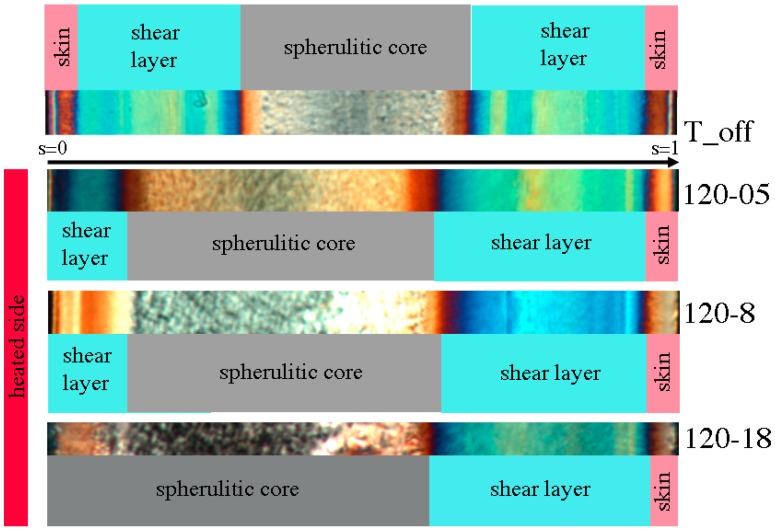
Optical micrographs of the samples from test runs T_off, 120-05, 120-8 and 120-18. The heated side is reported on the left most of the figure.

A preliminary analysis of morphology developed along the sample thickness allows observing some of the effects of an asymmetric temperature field. An example of cross-section morphology obtained by the optical micrograph of the sample T_off is shown at the top of Figure 4. The optical image in polarized light turns out to be symmetric for that sample and it is characterized by alternating colored bands. Each band is characteristic of a polymer layer with different levels of crystallinity and orientation. These layers were formed at different times of the process and thus experienced different thermomechanical histories before solidification. The competition between solidification and the relaxation process produces a characteristic distribution of orientation through the thickness of the sample. In the literature, the presence of three distinct morphologies is reported: a thin, oriented “skin layer” at the sample surface, formed by elongated structures at the sample wall and by less oriented “globular” elements soon after [12], an highly oriented fibrillar “shear layer”, just adjacent to the skin, and a less oriented layer, the “spherulitic” one, in the sample core [12,23,31,32].

The morphologies of the samples obtained keeping the cavity surface temperature at 120 °C for 0.5, 8 and 18 s are compared in Figure 4. The different layers developed along the sample thickness are strongly influenced by the temperature of the cavity surface. In particular, the shear layer moves toward the sample surface and its thickness decreases by increasing the heating time. The skin layer disappears for longer heating times, while with a heating time of 0.5 s the presence of the skin layer can not be clearly evidenced from the POM micrograph. Figure 4 shows that a 0.5 s heating time is sufficient to lose the symmetric morphology of the sample.

### 3.3. AFM Morphology

**Figure 5:**
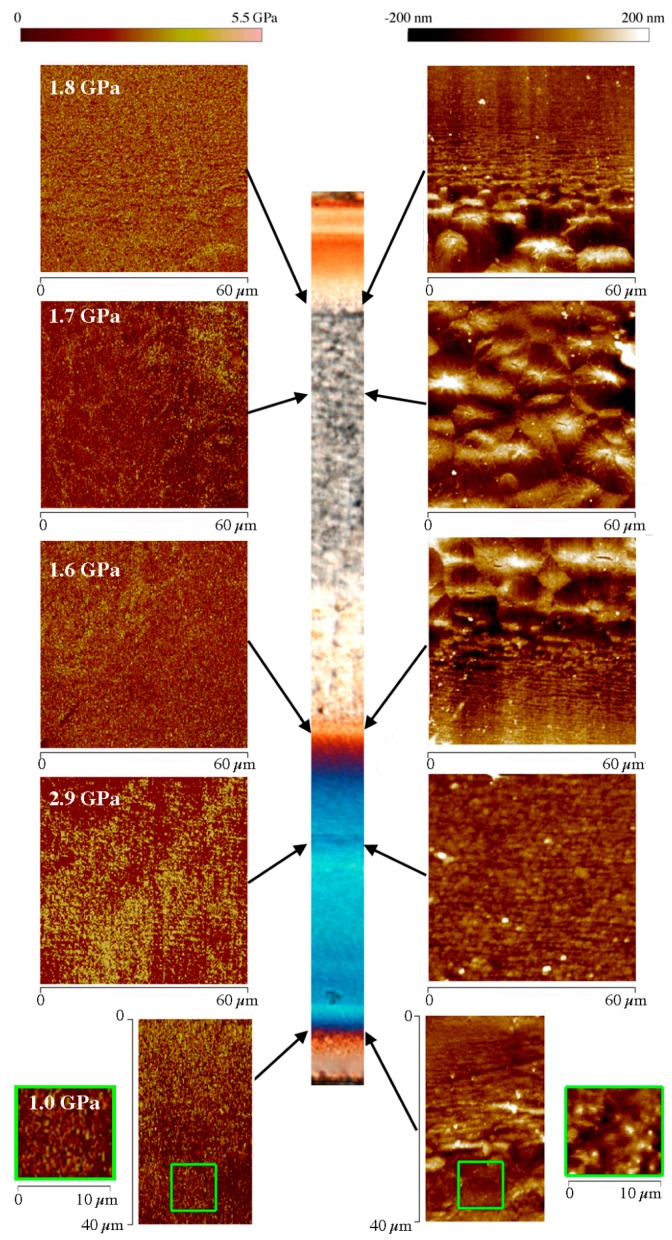
Atomic force microscopy (AFM) maps, height and modulus, obtained in different zones along the sample thickness, for the sample 120-8. The average moduli are reported in the map of each zone. Polarized Optical Microscopy (POM) micrographs and AFM maps have all the same alignment with flow direction in the thickness plane.

**Figure 6:**
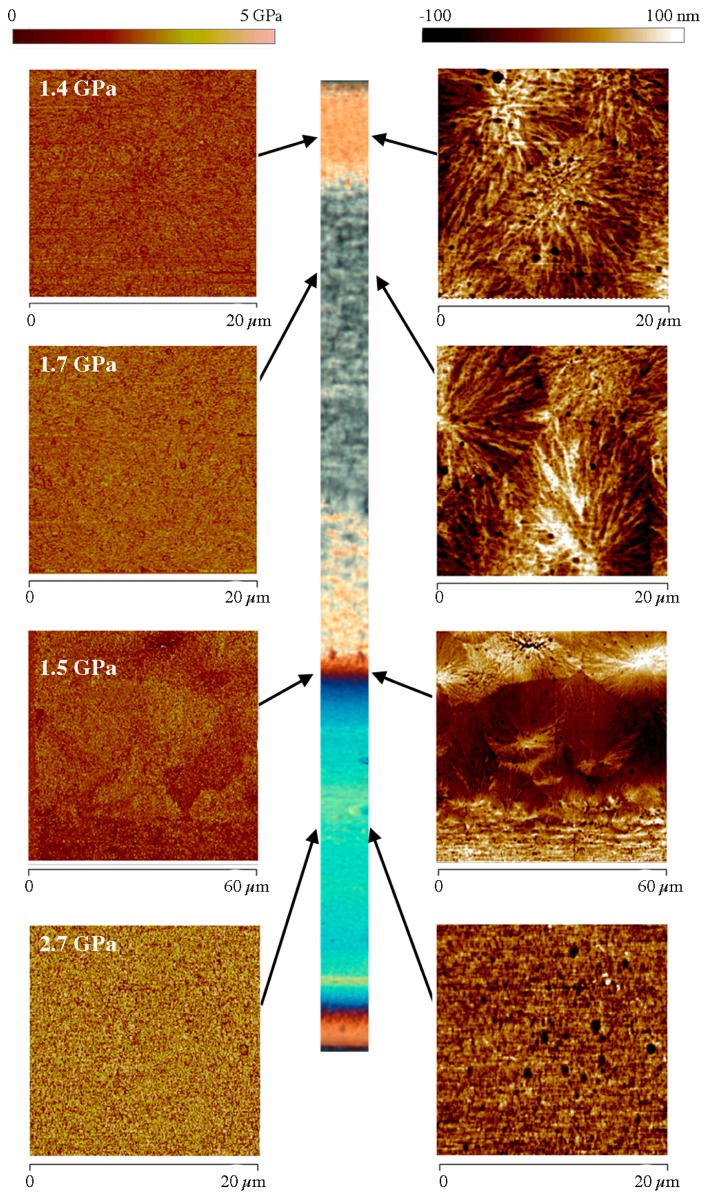
AFM maps, height and modulus, obtained in different zones along the sample thickness, for the sample 120-18. The average moduli are reported in the map of each zone. POM micrographs and AFM maps have all the same alignment with flow direction in the thickness plane.

A deeper investigation of the local morphology has been carried out by means of atomic force microscopy. Since HarmoniX AFM allows the elastic modulus on the sample surface to be mapped, it is possible to attempt a correlation between the morphology distribution and the mechanical properties along the sample thickness. The elastic modulus has been obtained from force-indentation curves applying the Derjaguis-Muller-Toporov (DMT) model [31]. Examples of the sample characterization are reported in Figure 5 and Figure 6. These figures show both the POM image of the whole thickness of the sample and some of the height and the elastic modulus maps acquired by HarmoniX AFM for the tests 120-8 and 120-18, respectively. The dimension of each AFM map has been selected to take into account the characteristic dimension of the morphological structures that are observed in the sample.

The micro structures developed in injection-molded parts are generally anisotropic and largely dependent on stretch distribution, molecular orientation, and cooling rate induced by the molding cycle. Comparing Figure 5 and Figure 6 one can observe that the morphology developed on the unheated side, where the temperature was at 28 °C, is essentially independent of the heating time. In particular, the AFM maps show that in the shear layer the fibrils are well packed with a mean thickness of about 250 ± 30 nm. On the heated side, the skin is not detectable for both selected heating times; the shear layer, detectable for the sample 120-8 of Figure 5, is made up of fibrils that have a mean thickness (500 ± 50 nm) that is bigger than the thickness of the fibrils on the unheated side. Figure 6, which refers to a longer heating time, shows that, on the heated side, the fibrils of the shear layer completely disappear and the spherulitic layer impinges on the sample surface. Evidently, in that case, the stretch due to the flow has sufficient time to relax the molecular orientation. Thus, the molecules crystalize into a spherulitic morphology during cooling to ambient temperature. Additionally, Figure 6 shows that there is a significant difference of the spherulite dimensions within the spherulitic layer. The spherulites that are formed close to the heated side appear smaller (15 μm diameter near the heated wall and 25 μm in the inner part of the layer). The reason for this difference can be due to the different cooling rates experienced by the melt when the heater is deactivated: close to the sample wall the cooling rate is higher; this favors higher nucleation density that means smaller spherulites. On the other border of the spherulitic layer, for both heating times, the spherulites appear elongated, consistent with the fact that they are in the transition zone. Finally, the morphology of the skin layer is shown by the AFM height map at the bottom of Figure 5. The fibrils of the shear layer tend to disappear and are replaced by un-oriented globular structures as shown by the magnification of the area enclosed in the green square reported in the AFM map.

Figure 5 and Figure 6 also show the elastic modulus maps acquired in the same positions where morphology was detected. The comparison among the elastic modulus maps shows that the shear layer on the unheated side is characterized by highest values of elastic modulus whereas the spherulitic layer shows the smaller values. The skin layer is characterized by the smallest modulus values (see Figure 5). This is consistent with the fact that in this layer globular elements have been found [12]. Moreover, the shear layer on the heated side is characterized by smaller modulus values with respect to the shear layer on the unheated side. This suggests that the fibrils found in these zones have a different mechanical behavior. In sample 120-18 (Figure 6) the spherulitic core impinges the sample wall on the heated side. The spherulites close to the heated side show modulus comparable with the elastic modulus of the spherulites in the inner part of the sample.

**Figure 7:**
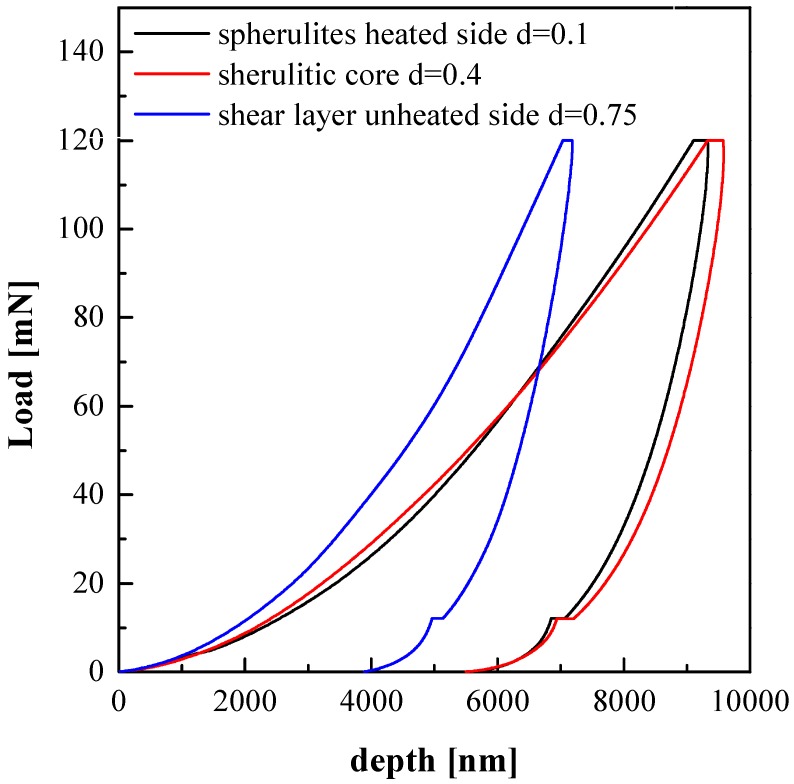
Load-displacement curves of three different positions along the thickness of sample 120-18.

For further investigation of mechanical modulus distributions, indentation tests have also been performed in different positions along the sample’s thickness. Figure 7 shows the load displacement curves obtained in different positions along the thickness of the sample 120-18. In particular, three different values of the dimensionless distance from the heated side, d, where considered: d = 0.1, d = 0.4 and d = 0.75. Following the Oliver–Pharr model [27], the elastic modulus mainly depends on the initial unloading slope in the load displacement plot. In particular, the elastic modulus increases with the unloading slope.

The indentation analyses confirm that the highest moduli were found in the shear layer on the unheated side, the spherulites show lower moduli.

**Figure 8:**
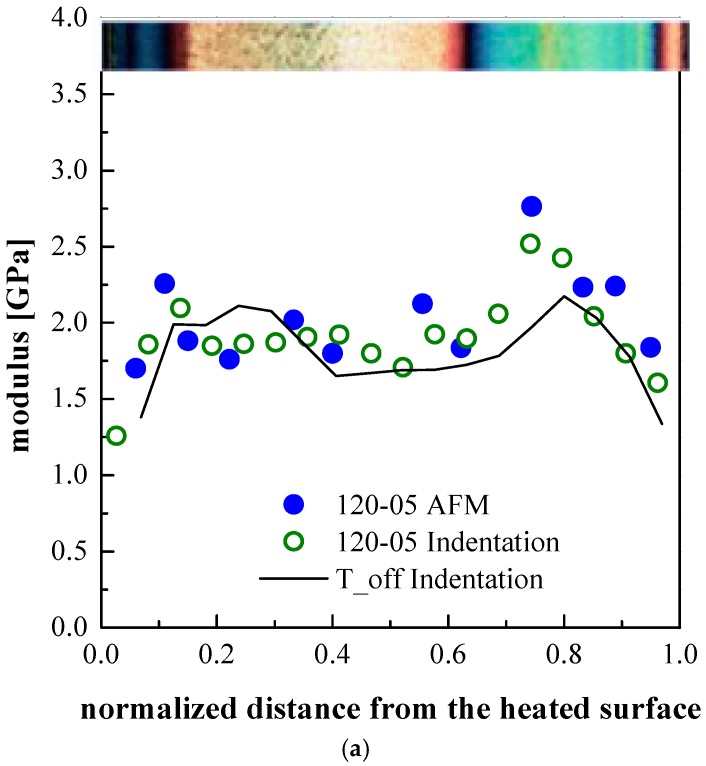

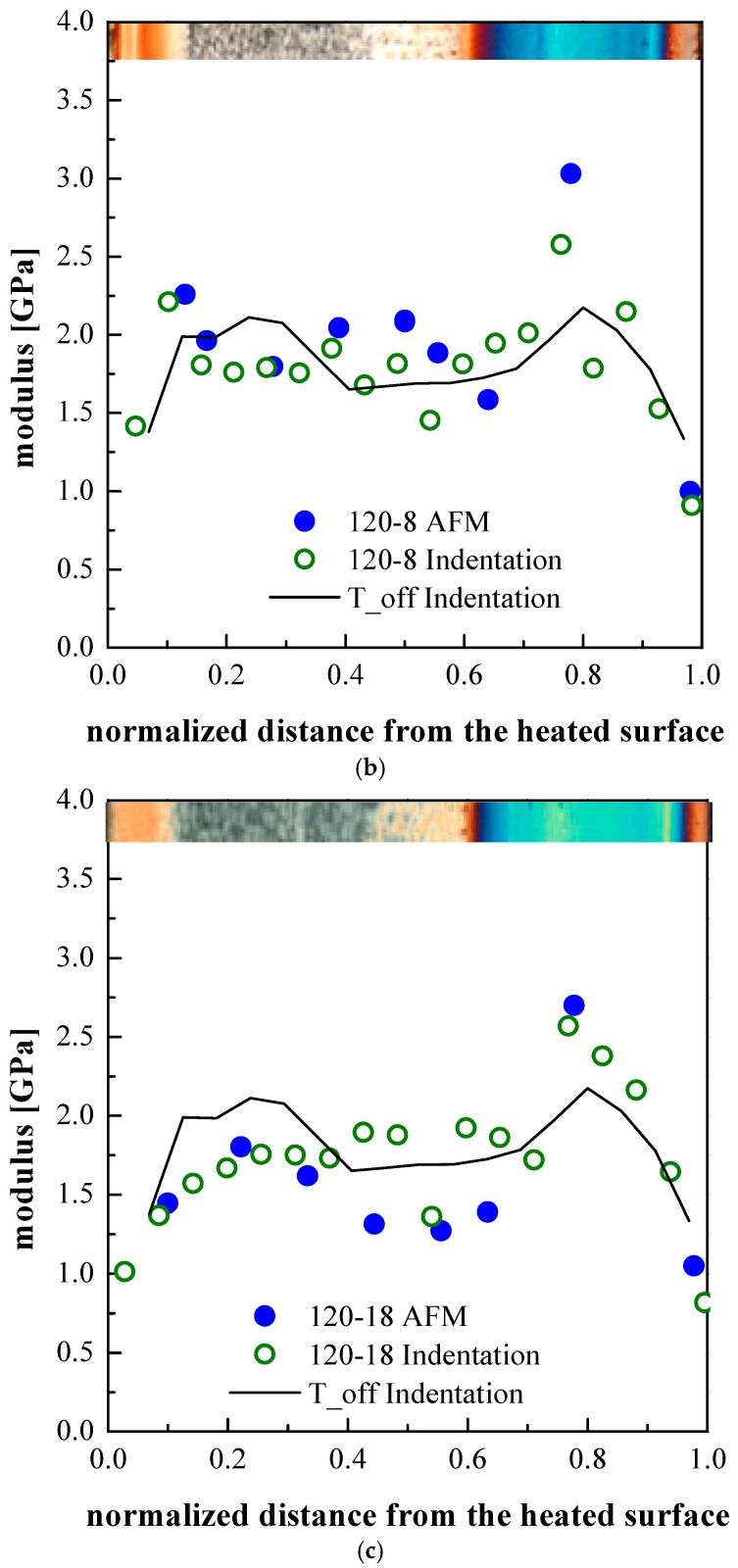
Distributions of elastic modulus along the sample thickness obtained by indentation and HarmoniX AFM tests for the samples (**a**) 120-05; (**b**) 120-8 and (**c**) 120-18.

Figure 8 shows the distributions of elastic moduli evaluated by both HarmoniX AFM and indentation analyses in several positions along the thickness of the samples 120-05, 120-8 and 120-18. In order to further study the effect of the cavity surface temperature on the mechanical properties of the samples, the experimental results obtained by the indentation analysis of the T_off sample are also reported in each plot.

The experimental results for the values of the modulus of the T_off sample reported in Figure 8 show a characteristic symmetric distribution with two maxima located in the shear layers, as shown in Figure 4. The skin layer shows the lowest moduli and the spherulitic core has intermediate modulus values. By increasing the mold temperature, the modulus distribution becomes asymmetric. This is due to both the uneven temperature field evolutions and to the asymmetrical flow developed during the filling stage. Because of the reduction of the shear layer thickness, on the heated side, the maximum value of the modulus approaches the sample surface. Moreover, the value of the maximum is lower with respect to the value of the maximum found on the unheated side. With 18 s heating time, the shear layer disappears on the heated side and the elastic modulus shows the characteristic values of the spherulitic layer.

**Figure 9:**
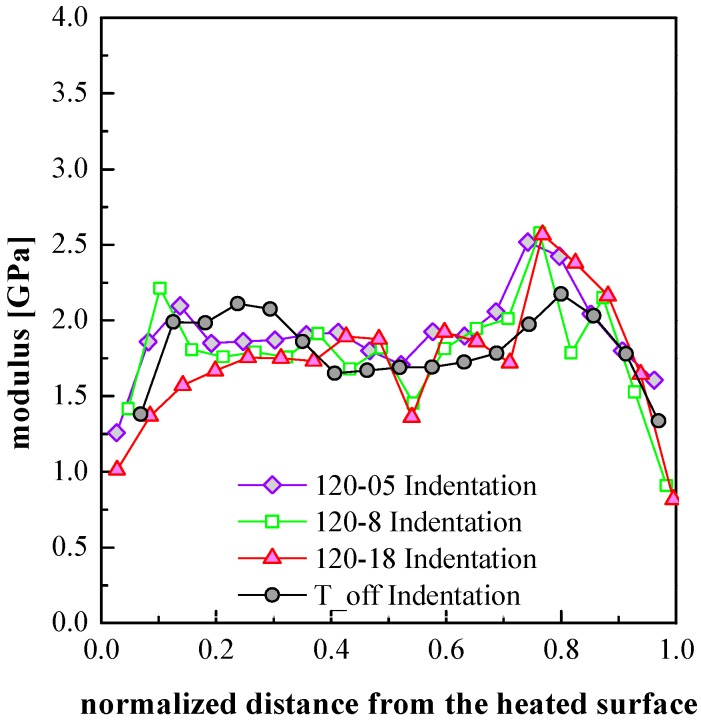
Elastic modulus distributions (obtained from indentation analyses) along the sample thickness for the samples T_off, 120-05, 120-8 and 120-18.

Figure 9 shows a comparison among the elastic modulus distribution obtained from indentation analyses of the samples T_off, 120-05, 120-8 and 120-18. On the unheated side the modulus appears significantly affected by the heating only close to the internal border of the shear layer; however, the modulus appears to be almost unaffected by the heating duration.

## 4. Discussion

The mechanisms that govern the temperature evolution inside the polymer part during the molding process are: fountain flow, heat convection, heat diffusion, viscous and latent heat generation [32]. Fountain flow occurs when the melt is forced, with elongational flow, outwards from the center of the flow front toward the mold wall. The fountain flow is recognized to be the main reason for the high level of molecular orientation found at the skin layer [33,34,35]. It is also responsible for a temperature profile that is quite uniform along the thickness of the flow front. This situation changes quickly soon after the first contact of the polymer with the surface by the effect of the heat transfer with the wall.

The evolution of temperature distribution inside an asymmetrically heated sample is given by Equation (1) [36], where convective contributions of the melt flow are not accounted for, or it could be neglected during the post-filling stages of the injection molding process. It considers a slab of thickness *s* starting from uniform temperature *T*_i_ (220 °C in the considered cases), with one surface held at constant temperature *T*_1_ and the other surface at *T*_2_ (120 °C and 28 °C, respectively).

(1) $$\frac{T\left(y,t\right)-T_{1}}{T_{2}-T_{1}}=\frac{y}{S}+\overset{\infty }{\underset{n=1}{\sum }}\frac{Z_{n}}{T_{2}-T_{1}}\left[exp\left(\frac{-\alpha n^{2}\pi^{2}t}{S^{2}}\right)*sin\left(\frac{n\pi y}{S}\right)\right]$$

where, α is the thermal diffusivity, *t* is the time and

(2) $$Z_{n}=2\frac{1-{\left(-1\right)}^{n}}{n\pi }\left(T_{i}-T_{1}\right)+\frac{2{\left(-1\right)}^{n}}{n\pi }\left(T_{2}-T_{1}\right)$$

In order to take into account the heat of crystallization, the thermal diffusivity *α* is corrected with the Stefan number, *Ste*, according to Equation (3).

(3) $$\alpha =\;\frac{\alpha_{1}}{1+Ste}$$

with:

(4) $$\alpha_{1}=\frac{k}{\rho \;C_{p}}\;Ste=\frac{X_{c}\cdot \Delta h_{\infty }}{C_{p}\cdot \left(T_{i}-T_{f}\right)}$$

where *Δh_∞_* is latent heat of crystallization, *X_c_* is the crystallinity and *T_f_* is the final temperature of the slab. For asymmetrical boundary conditions, *T_f_* is not constant along the slab thickness and depends on *s*. The values of the parameters adopted for the calculation are reported in Table 2.

**Figure 10:**
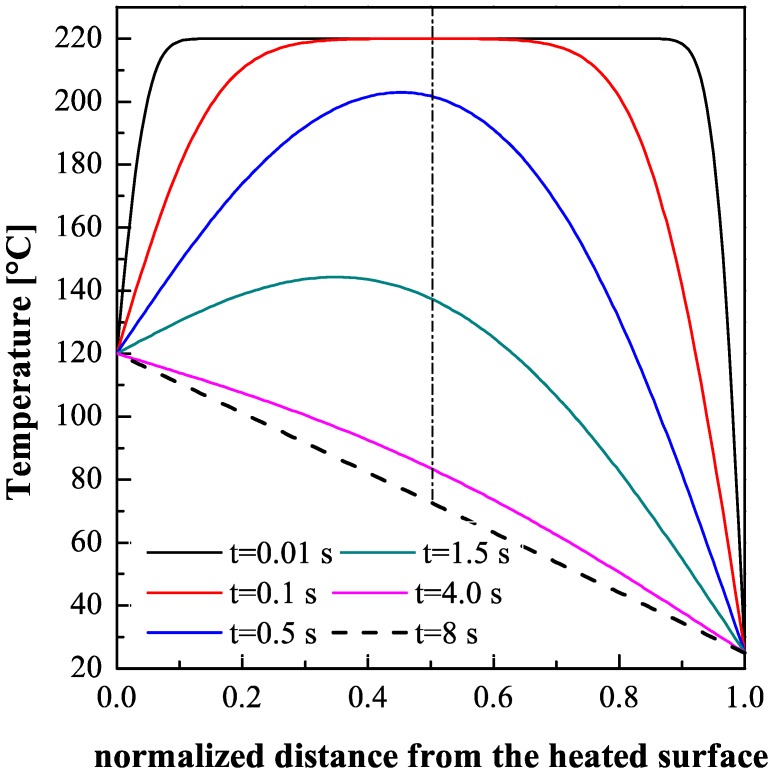
Predictions of temperature distribution along the cavity thickness at different times in an asymmetrically heated sample calculated by Equations (1)–(4).

The evolutions of the temperature distribution along the sample thickness, calculated by the Equations (1)–(4) at different times, are reported in Figure 10. The temperature distributions asymptotically approach the equilibrium distribution *T*_eq_ (the dotted line in Figure 10).

**Figure 11:**
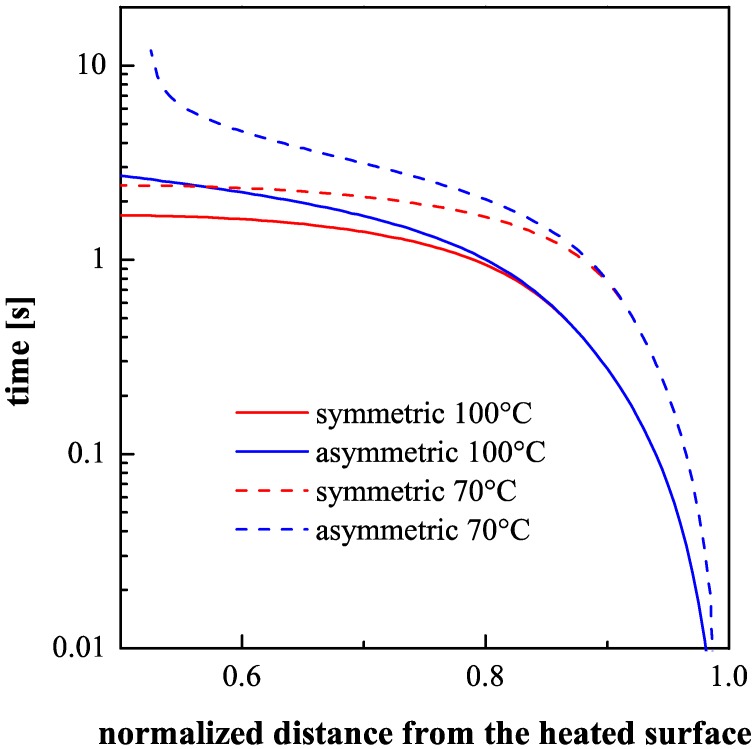
Distributions of the times taken to cool down to 100 °C and to 70 °C on the unheated side of the sample, calculated both for the T_off sample and for the asymmetrically heated sample.

Figure 11 shows the time to cool down each layer in the temperature range 70–100 °C, that corresponds to the temperature range within which the iPP crystallization takes place. The time distributions (down to 70 °C and to 100 °C) are reported for both symmetric thermal conditions and asymmetric thermal conditions.

The morphology distributions depend on the molecular stretch, which is determined, essentially, by the filling flow and on the subsequent relaxation time that, in turn, depends on the temperature evolution. As reported above, the surface heater was held active for three different times (namely, 0.5 s, 8 s and 18 s) after the contact of the melt with the cavity in position P2. The time 0.5 s corresponds to the cavity filling time, 18 s corresponds to the dominant relaxation time at 150 °C evaluated under processing conditions [37,38] and 8 s corresponds to about the time needed to reduce the distance from the equilibrium temperature distribution by about 95 percent.

The thicknesses of the shear layers on the side held at 28 °C show a small dependence upon the heating time (Figure 4). However, the asymmetric heating affects the evolution of temperature on the whole thickness. Figure 10 shows that there is a significant thickness (of about 0.15 mm) on the unheated mold surface where the cooling time results essentially unaffected by the mold heating on the other side (Figure 11). As expected, on this layer the morphologies of all samples correspond to that of the sample T_off, which experienced symmetrical cooling. Vice versa, the skin layer is not observed on the heated side where cooling rate, although high, does not achieve the values of the other side.

Deeper inside the unheated side, as shown in Figure 11, the cooling time during asymmetric heating experiments becomes larger with respect to the symmetrical cooling. The packing flow in the sample core, because of the higher temperature, remains active for a time longer than in the symmetrical case. Consequently, the molecular stretch reaches values sufficient to achieve shear layer thicknesses slightly wider than the thickness of the shear layer of the T_off case.

The micrographs reported in Figure 8 show that the morphology at the heated side changes with the heating time. In particular, with 0.5 s heating time a small shear layer was detected, which becomes thinner with 8 s and disappears with 18 s heating time. A parallel growth of the spherulitic layer toward the surface takes place due to surface heating. These effects must be determined by the gradual relaxation of the molecular stretch inside the polymer, while it is still in the molten state. As a consequence, it can be concluded that at the heated surface the polymer crystallizes during the fast cooling stages which follow the shutdown of the heating device.

The final mechanical properties are determined by the local structure, which obviously depends upon the local molecular stretch and by the local structuring achieved during solidification. The structuring from a melt (where the molecules are randomly distributed and deeply entangled) to a crystalline structured solid is a kinetic process and the structuring level enhances if more time is available. The time available to the polymer for crystalline structuring increases as the cooling rate (through the crystallization and related phenomena) decreases, thus for a given molecular stretch, structuring improves as the distance from the cold surface increases. On the other hand, the intensity of the molecular stretch (mainly generated by the shear intensity during the cavity filling) increases from the sample center toward the sample surface. Applying such a reason to the whole cross section, the moduli are expected to increase with the distance from the surface, due to the cooling rate decrease. At the same time, they are expected to decrease with the distance from the surface, due to the stretch decrease. The combination of these two factors is consistent with a maximum in between the symmetry plane and the sample surface, as it can actually be observed in the Figure 8 and Figure 9.

In the T_off conditions, the maximum within the shear layer is located closer to the spherulitic layer rather than to the external border of the shear layer. The structuring is expected to be very poor at both surfaces of the sample because of the extremely high cooling rate. On approaching the surfaces, the modulus decreases especially where skin layers are observed. When the heating stage is active the position of the maximum of the elastic modulus, on the unheated side, ends up being essentially unaffected by the heating stage.

What has still to be discussed is the effect of the heating time on the values of the maximum of the modulus within the shear layers. It is not a surprise that the moduli within the two shear layers on the heated side (of the samples 120-05 and 120-8) follow similar paths (with clear small maxima) since both of those layers crystallize during the fast cooling which follows the shutdown of the heating device. The sample 120-18 does not show any maximum on the heated side since in this case the polymer chains have enough time to relax and the shear layer is not observed.

On the unheated side, it has to be considered that the three samples (120-05, 120-8, 120-18) experienced cooling histories that are very different one from the other. Furthermore, within each test, the cooling histories through the crystallization are a function of the distance from the surface, also within the shear layer. The temperature evolutions within the sample shown in Figure 10 were calculated under the condition that the temperature on the heated side is held at 120 °C. However, for the sample 120-05 the heating was interrupted soon after the end of the filling, i.e., 0.5 s. At that time, the temperature distribution on the sample cross-section was already significantly different with respect to the symmetrical cooling case (see the temperature distribution at *t* = 0.5 s, in Figure 10). Furthermore, the sample surface will take a small but significant time to decrease to 28 °C, as shown in Figure 2. As a consequence, the subsequent cooling through the crystallization on the unheated side will be slowed down. This determines a larger time for structuring and thus it justifies the increase (with respect to the symmetric case) of the maximum of the modulus inside the shear layer.

The other two samples, 120-8 and 120-18, undergo a first cooling toward the temperature of the dotted line in Figure 10, and afterward their temperature remains essentially constant until the heating shutdown and then they undergo a second cooling to the mold temperature after the heating shutdown. At the end of the first cooling step the temperature becomes sufficiently small (45–60 °C at 0.6–0.8 mm distance from the heated wall) to assure that, in both conditions, the polymer crystallizes in the time available.

It is well known that crystallization temperature during cooling decreases with the increase of the cooling rate and increases by the effect of the flow, both effects increase toward the sample surface and partially compensate each other. The crystallization of the α-phase of polypropylene has a maximum rate at about 70 °C with a half crystallization time of about 2 s [39,40]. That time decreases of 1–2 orders of magnitude by the effect of the flow, as a consequence the α crystallization temperature in a shear layer has to take place in the interval 70–100 °C. The permanence time within that temperature interval determines the time available, during cooling, for structuring during solidification, which in its turn (for a given stretch) determines the material properties (the modulus in our case). The permanence times within that temperature interval can be evaluated from Figure 11 also for the sample T_off. In particular, Figure 11 refers only to the unheated side. The permanence times are only slightly dependent on the distance from the sample surface in the shear layer. However, in the internal zone of the shear layer, the permanence times in the crystallization temperature range for the samples 120-8 and 120-18 show values clearly larger than those calculated for the sample T_off. This is consistent with the findings related to the mechanical properties: the sample obtained with 120 °C mold surface temperature shows higher maximum values of elastic modulus in the shear layer of the unheated side with respect the shear layer of the T_off sample.

## 5. Conclusions

In this work, iPP samples were obtained by injection molding adopting a cavity with a surface kept at a high temperature for different time periods (0.5 s, 8 s and 18 s) during the process. Resulting molded samples have been mechanically characterized at different length-scales by indentation and AFM HarmoniX.

The asymmetry of the thermal conditions on the mold surface completely changes the classical skin-core structure shown by the samples. The three characteristic morphological layers, namely skin, shear and core layer are affected in a different way by external heating. The core layer, generally characterized by a spherulitic morphology with intermediate modulus, increases in thickness and moves up to the heated sample side. The shear layers show different behaviors with respect to the half-thickness position. The one closer to the heated side moves in the surface direction and gradually decreases its thickness up to completely disappear for 18 s heating time. On the opposite, the layer closer to the unheated surface slightly increases in dimension with respect to those formed in a symmetrically cooled sample. The skin layer, characterized by loosely structured crystalline elements, is practically unaffected on the unheated side, yet it disappears even for a very short heating time (0.5 s) on the heated side.

Higher values of the modulus were found in the shear layers (especially those close to the unheated side). Minimum and intermediate values were found in the skin and in the spherulitic layers, respectively.

The variations of the modulus were interpreted on the basis of variations of both the molecular stretch and the time available for molecular structuring: the cooling rate through the crystallization determines the time available and thus affects the level of structuring achieved inside the solid. The modulus increases with the increase of local structuring and molecular stretch.

## Figures and Tables

**Table 1:** Experimental molding conditions.

Test Run	*P*_e_ (W/cm^2^)	*T*_level_ (°C)	*t*_h_ (s)	*t*_a_ (s)	*P*_hold_ (bar)
T_off	0	28	0	0	300
120-05	7	120	0.5	2	300
120-8	7	120	8	2	300
120-18	7	120	18	2	300

**Table 2:** Parameters adopted to calculate the temperature distribution along the sample thickness.

Parameter	Value
*α*_1_	8.25 × 10^−8^ m^2^·s^−1^
*C*_p_	2.62 J·g^−1^·K^−1^
*Δh_∞_*	114 J·g^−1^
*X*_c_	0.5
*T*_f_ at midplane	70 °C
